# Maturation of the malarial phosphatidylserine decarboxylase is mediated by high affinity binding to anionic phospholipids

**DOI:** 10.1016/j.jbc.2023.104659

**Published:** 2023-03-29

**Authors:** Jae-Yeon Choi, Lauren Lopes, Choukri Ben Mamoun, Dennis R. Voelker

**Affiliations:** 1 Basic Science Section, Department of Medicine, National Jewish Health, Denver, Colorado, USA; 2 Department of Internal Medicine, Section of Infectious Diseases, Yale School of Medicine, New Haven, Connecticut, USA

**Keywords:** phosphatidylserine decarboxylase, phosphatidylserine, phosphatidylethanolamine, proenzyme processing, enzyme regulation, anionic phospholipid, phosphatidylglycerol, phosphatidic acid, cardiolipin, phosphatidylcholine, liposome, solid phase binding, surface plasmon resonance

## Abstract

Decarboxylation of phosphatidylserine (PS) to form phosphatidylethanolamine by PS decarboxylases (PSDs) is an essential process in most eukaryotes. Processing of a malarial PSD proenzyme into its active *alpha* and *beta* subunits is by an autoendoproteolytic mechanism regulated by anionic phospholipids, with PS serving as an activator and phosphatidylglycerol (PG), phosphatidylinositol, and phosphatidic acid acting as inhibitors. The biophysical mechanism underlying this regulation remains unknown. We used solid phase lipid binding, liposome-binding assays, and surface plasmon resonance to examine the binding specificity of a processing-deficient *Plasmodium* PSD (PkPSDS308A) mutant enzyme and demonstrated that the PSD proenzyme binds strongly to PS and PG but not to phosphatidylethanolamine and phosphatidylcholine. The equilibrium dissociation constants (K_d_) of PkPSD with PS and PG were 80.4 nM and 66.4 nM, respectively. The interaction of PSD with PS is inhibited by calcium, suggesting that the binding mechanism involves ionic interactions. *In vitro* processing of WT PkPSD proenzyme was also inhibited by calcium, consistent with the conclusion that PS binding to PkPSD through ionic interactions is required for the proenzyme processing. Peptide mapping identified polybasic amino acid motifs in the proenzyme responsible for binding to PS. Altogether, the data demonstrate that malarial PSD maturation is regulated through a strong physical association between PkPSD proenzyme and anionic lipids. Inhibition of the specific interaction between the proenzyme and the lipids can provide a novel mechanism to disrupt PSD enzyme activity, which has been suggested as a target for antimicrobials, and anticancer therapies.

Phosphatidylserine decarboxylases (PSD) are important enzymes in phospholipid synthesis and membrane biogenesis in bacteria, yeast, protozoa, plants, and animals (1, 2, 3, 4). PSDs catalyze the decarboxylation of phosphatidylserine (PS) to form phosphatidylethanolamine (PE) (5, 6, 7, 8), an essential structural phospholipid found in the membranes of diverse organisms (5, 8, 9, 10, 11, 12). PE can be formed not only by PSD but also *via* other routes in various organisms (4). These routes include *de novo* synthesis from ethanolamine, through the CDP–ethanolamine pathway, in most lower and higher eukaryotes (also called the Kennedy pathways) (13); polar head group exchanges among the amine containing polar headgroups of PS, phosphatidylcholine (PC), and PE in mammals (4); and acylation of lyso-PE (14, 15). Although PE can be produced by different routes in eukaryotes, PSD-dependent PE formation in mitochondria is essential for the function of the organelle and fitness and viability of multiple organisms (9, 12, 16, 17, 18). In the yeast *Saccharomyces cerevisiae,* ablation of the *PSD1* gene, which encodes a mitochondrial PSD enzyme, results in ethanolamine auxotrophy on nonfermentable carbon sources and mitochondrial instability (12). In mice, inactivation of the *PSD* gene results in embryonic lethality with structural and functional defects of the mitochondria (9). In humans, mutations that affect expression or function of PSD activity have been linked to a rare form of dwarfism, with patient fibroblasts displaying mitochondrial abnormality (19). Very recently, PSD activities have been implicated in cancer development, and targeting PSD has been proposed to treat certain types of cancer. Keckesova *et al* showed that the mitochondrial serine *beta*-lactamase-like protein (LACTB) acts as a tumor suppressor that inhibits the proliferation of certain breast cancer cells through inhibition of mitochondrial lipid biosynthesis, by decreasing the PSD enzyme levels (by 60–95%) in mitochondria (20). Increasing PS through inhibition of PSD was suggested as a potential therapeutic strategy for acute myeloid leukemia (AML) (21, 22). Inhibition of human PSD (PiSD) by MMV007284, an inhibitor of the malarial PSD enzyme (8), resulted in decreased AML stemness and increased AML differentiation, without affecting normal hematopoiesis (22).

PSDs are unusual enzymes that utilize a pyruvoyl prosthetic group for catalysis (23, 24, 25). The pyruvoyl moiety is created in a concerted reaction that occurs within a consensus GS∗S/T sequence present in the proenzyme (1, 24). The proteolytic cleavage in the *Plasmodium knowlesi* PSD proenzyme occurs between 307G and 308S∗ which creates a mature enzyme consisting of a large β-subunit derived from the N-terminal PSD proenzyme and a small α-subunit derived from the C-terminal region of the PSD proenzyme. The α-subunit harbors an N-terminal pyruvoyl moiety which is derived from 308S∗ (26). The pyruvoyl prosthetic group is the crucial component of the active site of the PSD enzyme. It forms a Schiff base intermediate with the PS substrate, which is essential for, and drives, the catalytic reaction (23, 27).

The proteolytic cleavage activity is executed by the PSD proenzyme, itself, which belongs to a class of serine proteases. PMSF, a serine protease inhibitor, inhibits PkPSD processing (26). The PkPSD contains conserved aspartic acid (D139), histidine (H198), and serine (S308) residues, which form a canonical D-H-S active site of a serine protease (26). Site-directed mutagenesis of Asp-139 or His-198 or Ser-308 to alanine results in a complete loss of endoproteolytic processing of the PkPSD proenzyme and ultimately loss of PSD enzyme activity (26). The essential role of the catalytic triad was also confirmed in *S. cerevisiae* PSD1 where a conserved histidine (His345), Asp210, and Ser463 forms a classic Ser-His-Asp catalytic triad (28). Proteolytic activity of the PkPSD proenzyme only occurs in *cis*, meaning that each molecule executes self-cleavage (26). Previous research showed that anionic phospholipids regulated *in vitro* maturation of PkPSD proenzyme into *α* and *β* subunit (3). PS, an anionic phospholipid, increased the extent of maturation and the final PSD enzyme activity of PkPSD produced by an *in vitro* transcription and translation reaction, whereas the zwitterionic phospholipids, PC and PE, had no influence on the maturation process (Fig. 1). Interestingly, the process was inhibited by other anionic phospholipids, such as phosphatidic acid (PA), phosphatidylglycerol (PG), and phosphatidylinositol (PI) (3). A third anionic phospholipid, cardiolipin, which is a major phospholipid in mitochondrial outer membranes, also inhibited the processing of PkPSD (unpublished data).

**Figure 1:**
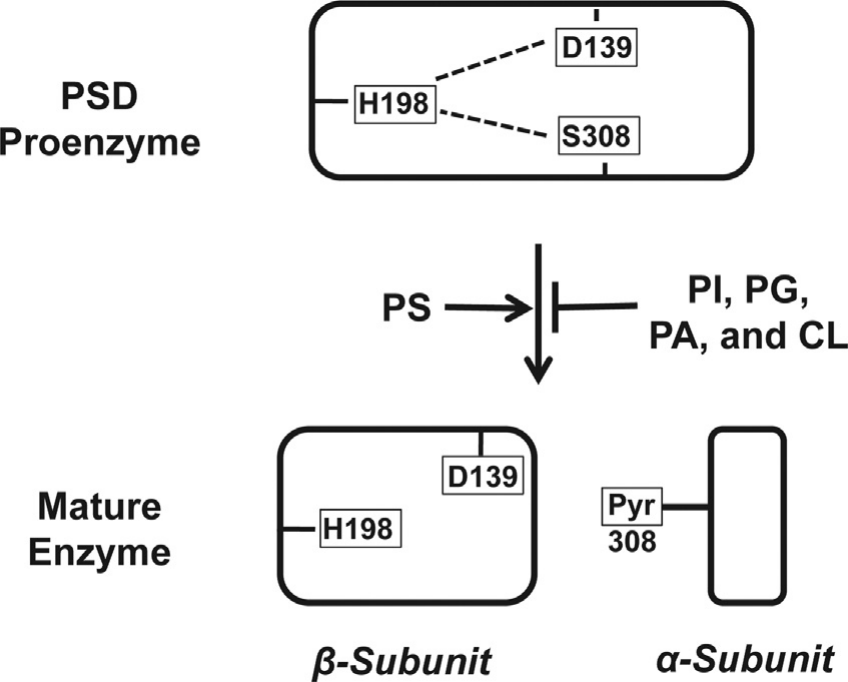
**Schematic diagram of PSD processing and its regulation by anionic phospholipids.** PkPSD is synthesized as an inactive proenzyme. Autoendoproteolytic cleavage of the proenzyme requires the D,H,S catalytic triad and leads to active PSD enzyme composed of *α* and *β* subunits. During the cleavage process, serine within the cleavage site is converted to pyruvate. *In vitro* processing of the PkPSD is enhanced by the anionic phospholipid, PS, but inhibited by other anionic phospholipids, such as phosphatidylglycerol (PG), phosphatidylinositol (PI), phosphatidic acid (PA), or cardiolipin (CL). Pk, *Plasmodium knowlesi*; PS, phosphatidylserine; PSD, PS decarboxylase.

In this report, we present data demonstrating that in order for the malarial PSD proenzyme to be processed into a mature enzyme, it has to physically interact with the PS lipid, the activator of the PSD maturation. The physical interactions between PS and the proenzyme were determined by solid phase binding, liposome cosedimentation assays, and surface plasmon resonance (SPR).

## Results

### PS and PG bind to PkPSD proenzyme

**Figure 2:**
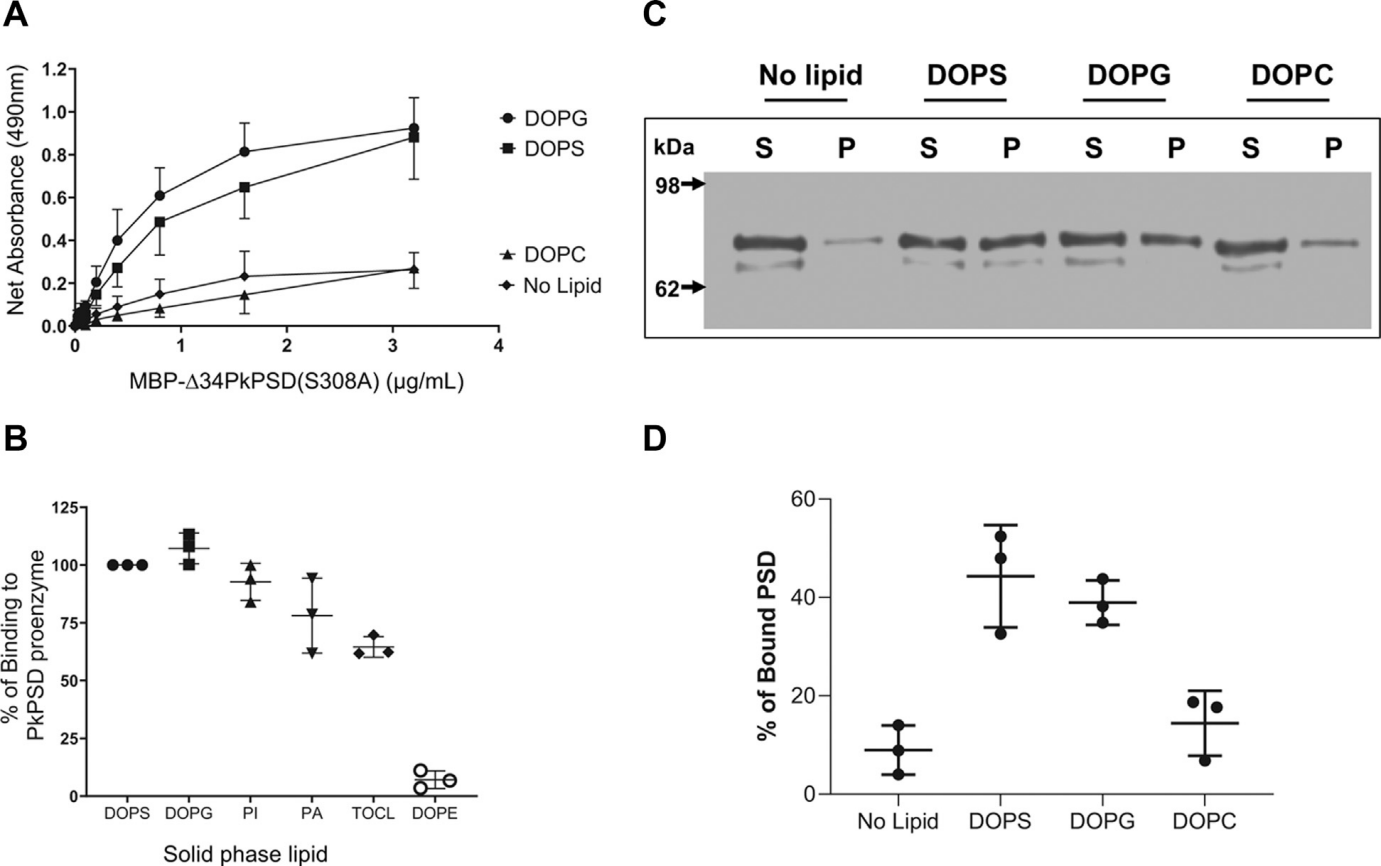
**PkPSD proenzyme binds to solid phase anionic lipids.***A*, a stable form of the PkPSD (MBP-Δ34PkPSD(S308)) proenzyme shows saturable binding to solid phase PS and PG adsorbed onto microtiter wells, as described in Experimental procedures. PkPSD proenzymes (MBP -Δ34PkPSD(S308A)) in a range of 0 to 3.4 μg/ml were incubated with lipids precoated onto a 96-well plate at 37 °C for an hour. *B*, 3.4 μg/ml of PkPSD proenzyme was incubated with various classes of phospholipids (DOPG, DOPS, PI, PA, TOCL, and DOPE) immobilized on the wells of the 96-well plate. PSDs bound to the immobilized lipids were detected by ELISA using antibodies recognizing the MBP fusion partner, and goat HRP-conjugated anti mouse antibody, and quantified by measuring absorbance at 490 nm as described in Experimental procedures. The data are from six (for *A*) or three (for *B*) independent experiments, and values shown are means ± SD. Either + or – SD values are shown for each point in fig. *A*. *C*, multilamellar liposome cosedimentation assays were performed with affinity-purified MBP-Δ34PkPSD(S308) (6.25 μg/ml) and freshly prepared multilamellar liposomes, at 37 °C. After 45 min, the mixtures were centrifuged at 10,000*g* for 5 min, at 4 °C. The supernatants were carefully transferred to a fresh tube, and the liposome pellets were resuspended in a defined volume (0.25 ml) of buffer. Proteins in both fractions were analyzed by SDS-PAGE, followed by Western blotting, using primary mouse anti-MBP antibody, and secondary goat HRP-conjugated anti-mouse Ig antibody. *D*, % of liposome-associated PSDs are shown after image J quantification of the western blots. DOPC, dioleoyl phosphatidylcholine; DOPE, dioleoyl phosphatidylethanolamine; DOPG, dioleoyl phosphatidylglycerol; DOPS, dioleoyl phosphatidylserine; MBP, maltose binding protein; PA, phosphatidic acid; PG, phosphatidylglycerol; PI, phosphatidylinositol; Pk, *Plasmodium knowlesi*; PSD, PS decarboxylase; TOCL, tetraoleoyl cardiolipin.

To understand the regulatory mechanisms of the PkPSD maturation by anionic phospholipids, we examined if the regulation occurred through direct physical interactions between the PkPSD proenzyme and the anionic phospholipids. Since PkPSD proenzyme expressed in *Escherichia coli* host cells tends to be readily processed into a mature enzyme under both *in vivo* and *in vitro* conditions, we used a stable form of a proenzyme, PkPSD(S308A) which harbors a mutation in S308, which is the amino acid eventually converted to pyruvate, to generate the active site of mature PSD. Not only does S308A prevent generation of the active site of the mature enzyme, but it also prevents cleavage of the proenzyme into mature α and β subunits (26). First, we performed a solid phase binding analysis using an affinity purified, chimeric recombinant PSD enzyme, (MBP-His_6_Δ34PkPSD(S308A)) to test the interaction between the proenzyme and the solid phase anionic lipids. The PSD(S308A) bound to the lipid was detected and quantified by ELISA using an anti-MBP antibody. The data shown in Figure 2*A* demonstrate that the PkPSD proenzyme has a strong affinity toward the anionic phospholipids, PS and PG, coated onto the 96-well plates, whereas it has poor affinity toward mock (ethanol treated) or PC-coated solid phase wells. PSD proenzyme binding to solid phase PS- and PG-coated wells was proenzyme concentration dependent (Fig. 2*A*). The PkPSD proenzyme also binds to other anionic phospholipids, such as tetraoleoyl cardiolipin, PA, and PI, but not dioleoyl-PE (Fig. 2*B*). PSD binding to PS and PG lipids was also confirmed by a multilamellar liposome-binding assay (Fig. 2*C*). After incubation of PSD proenzyme with multilamellar liposomes, the lipid and PSD mixtures were sedimented by centrifugation at 10,000*g* for 10 min. After the centrifugation, the multilamellar liposomes and the liposome-bound PSD are sedimented into a pellet, whereas unbound PSD proenzymes remain in the supernatant. Figure 2*C* shows the unbound (supernatant) and bound PSD (pellet) proenzymes detected by Western blot analysis. Strong signals are shown among the liposome pellets, for the PSD proenzymes preincubated with PS or PG liposomes, whereas those incubated with mock (no lipid) and PC liposomes were mostly found in the supernatant, as shown in Figure 2, *C* and *D*. The detected PSD protein band intensities quantified by imageJ software are shown in Figure 2*D*. Approximately 40% of PSDs were associated with the PS or PG liposomes, whereas only ∼10% of PSDs were cocentrifuged either with PC liposomes, or no liposomes. Taken together, the data from solid phase binding and liposome sedimentation assays show that PSD proenzymes bind strongly and stably to the anionic lipids.

### Binding kinetics of PSD proenzyme interactions with anionic lipids

**Figure 3:**
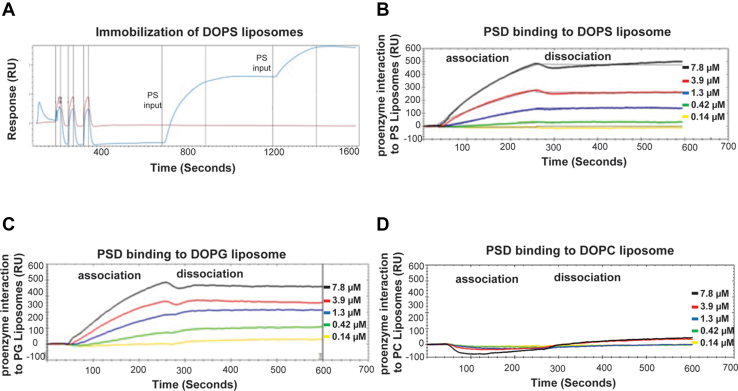
**Binding kinetic analysis of purified MBP-Δ34PkPSD(S308A) interactions with immobilized PS and PG.** Binding kinetic studies were performed by SPR analysis using liposomes immobilized on the LIP-1 sensor chip (DOPS, DOPG, or DOPC liposomes at 0.5 mg/ml). MBP-Δ34PkPSD(S308A) at varied concentrations (0−7.8 μM) were injected at a flow rate of 20 μl/min. The association and dissociation reactions occur for 240 and 360 S, respectively. *Panel A* shows the PS liposome immobilization. The association of the MBP-Δ34PkPSD(S308A) with the immobilized PS (*panel B*), immobilized PG (*panel C*), or immobilized PC (*panel D*) are presented. Each panel represents a single experiment from at least three independent tests. The binding kinetics data were collected and analyzed using a simple 1:1 interaction model and TraceDrawer software (Nicoya). MBP, maltose binding protein; PC, phosphatidylcholine; PG, phosphatidylglycerol; Pk, *Plasmodium knowlesi*; PS, phosphatidylserine; PSD, PS decarboxylase; SPR, surface plasmon resonance.

To further analyze molecular interaction of the PSD proenzyme and the anionic phospholipids, SPR experiments were performed. Figure 3*A* shows that the DOPS liposomes were immobilized on the sensor chip after two successive injections. Association (first 240 s) and dissociation (next 360 s) reactions of the PSD proenzymes (240 s) with the immobilized liposomes were performed. Figure 3, *B* and *C* show that binding increases with increasing concentration of proenzyme for both immobilized PS (Fig. 3*B*) and immobilized PG (Fig. 3*C*). In contrast, the proenzyme fails to bind the immobilized PC as shown in Figure 3*D*. The binding kinetics between the liposomes and the PSD proenzyme were analyzed by the TraceDrawer software (from Nicoya) following a 1:1 Langmuir model (29). The equilibrium dissociation constants (K_d_) of PkPSD with DOPS and DOPG liposomes were 80.4 nM and 66.4 nM, respectively. The data indicate that binding of the DOPS and DOPG to the PSD proenzymes shows similar affinity.

### PS and PG bind to the same locus of the PkPSD proenzyme

**Figure 4:**
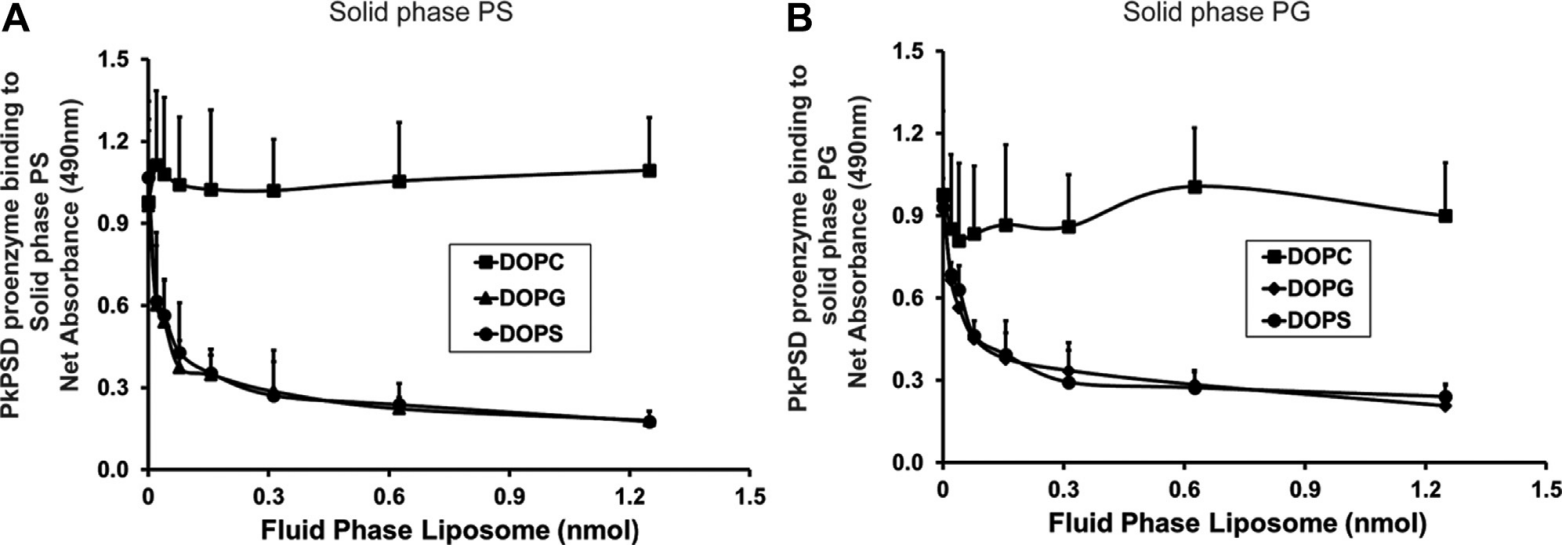
**Fluid phase PS and PG compete for PSD proenzyme binding.** PkPSD proenzyme binds to solid phase PS or PG in the presence of liposomes. PkPSD proenzymes (MBP-Δ34PkPSD(S308A)) at 3.4 μg/ml were incubated for an hour with solid phase PS (*A*) or PG (*B*) precoated on the 96-well plate at 37 °C as described in Figure 2. For this test, various concentrations of fluid phase liposomes consisting of DOPS, DOPG, or DOPC (0–1.25 nmol) were also added to the well to measure fluid phase lipid competition with solid phase lipids for PSD binding. Fluid phase liposome-bound PSDs were washed away and only the solid phase lipid-bound PSDs were detected by ELISA assay and quantified by measuring absorbance at 490 nm. The data are from three independent experiments, values are Means + SD. MBP, maltose binding protein; PG, phosphatidylglycerol; Pk, *Plasmodium knowlesi*; PS, phosphatidylserine; PSD, PS decarboxylase.

Next, we investigated whether the activator and the inhibitor of the PSD processing share a common binding site in the PSD proenzyme. We tested if PSD proenzyme, MBP-Δ34PkPSD(S308A), binding to the solid phase lipid as either PS (activator) or PG (inhibitor) can be inhibited by fluid phase liposomes. Various concentrations of fluid phase liposomes composed of either DOPS, DOPG, or DOPC were added at the same time as PSD proenzyme addition to the wells of a 96-well plate that were precoated with either DOPS or DOPG. If the fluid phase liposomes compete with solid phase lipids for the PSD binding, the final readout of solid phase lipid binding by the proenzyme will be lowered. The data in Figure 4 show that 1.2 nmoles of solid phase DOPS-bound PSD proenzymes were reduced as much as 83.5 or 81.3 % by 1.2 nmoles of fluid phase liposomes consisting of DOPS or DOPG, respectively, but increased 11.6% by those of DOPC (Fig. 4*A*). Similarly, solid phase DOPG-bound PSD proenzymes were reduced as much as 74.1 or 78.3 % by 1.2 nmoles of fluid phase liposomes consisting of DOPS or DOPG, respectively, but increased 5.3 % by DOPC (Fig. 4*B*). The data demonstrate that binding of PSD proenzyme to the solid phase PS (Fig. 4*A*) and PG (Fig. 4*B*) was similarly inhibited by the increasing dose of both fluid phase PS and PG liposomes, but not by fluid phase PC liposomes, and that the PSD proenzyme prefers fluid phase liposomes over the solid phase lipids as binding partners.

### Ionic interactions govern the anionic lipid binding with the PSD proenzyme

**Figure 5:**
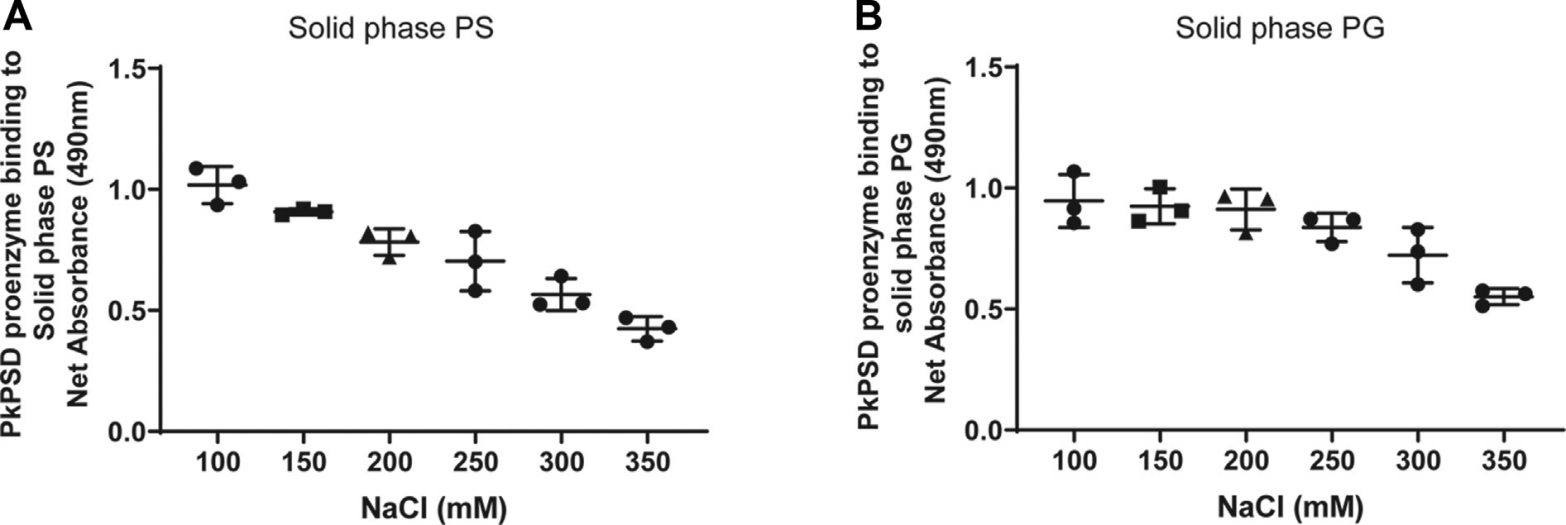
**Interactions of PS and PG with PSD proenzyme are disrupted by high salt concentrations.** 3.4 μg/ml of PkPSD proenzymes were incubated with solid phase PS (*A*) or PG (*B*) as described in Figure 2. This binding assay, however, was conducted in a buffer containing various concentrations of NaCl (0–350 mM). The data are from three independent experiments, and values are Means ± SD. PG, phosphatidylglycerol; Pk, *Plasmodium knowlesi*; PS, phosphatidylserine; PSD, PS decarboxylase.

Since multiple negatively charged lipids were found to interact with the PSD proenzyme, we tested if the ionic interaction is the main driver of the lipid binding to the PSD protein. It has been reported that binding through ionic interaction can be disrupted when the binding partners are subjected to high ionic strength conditions (30, 31). The data in Figure 5 show that the interactions were affected by buffers that contained high concentrations of NaCl. The solid phase PS- or PG-bound PkPSD proenzymes were reduced by 58.6 % or 41.8%, respectively, in buffers containing 350 mM NaCl compared to those of 100 mM NaCl.

**Figure 6:**
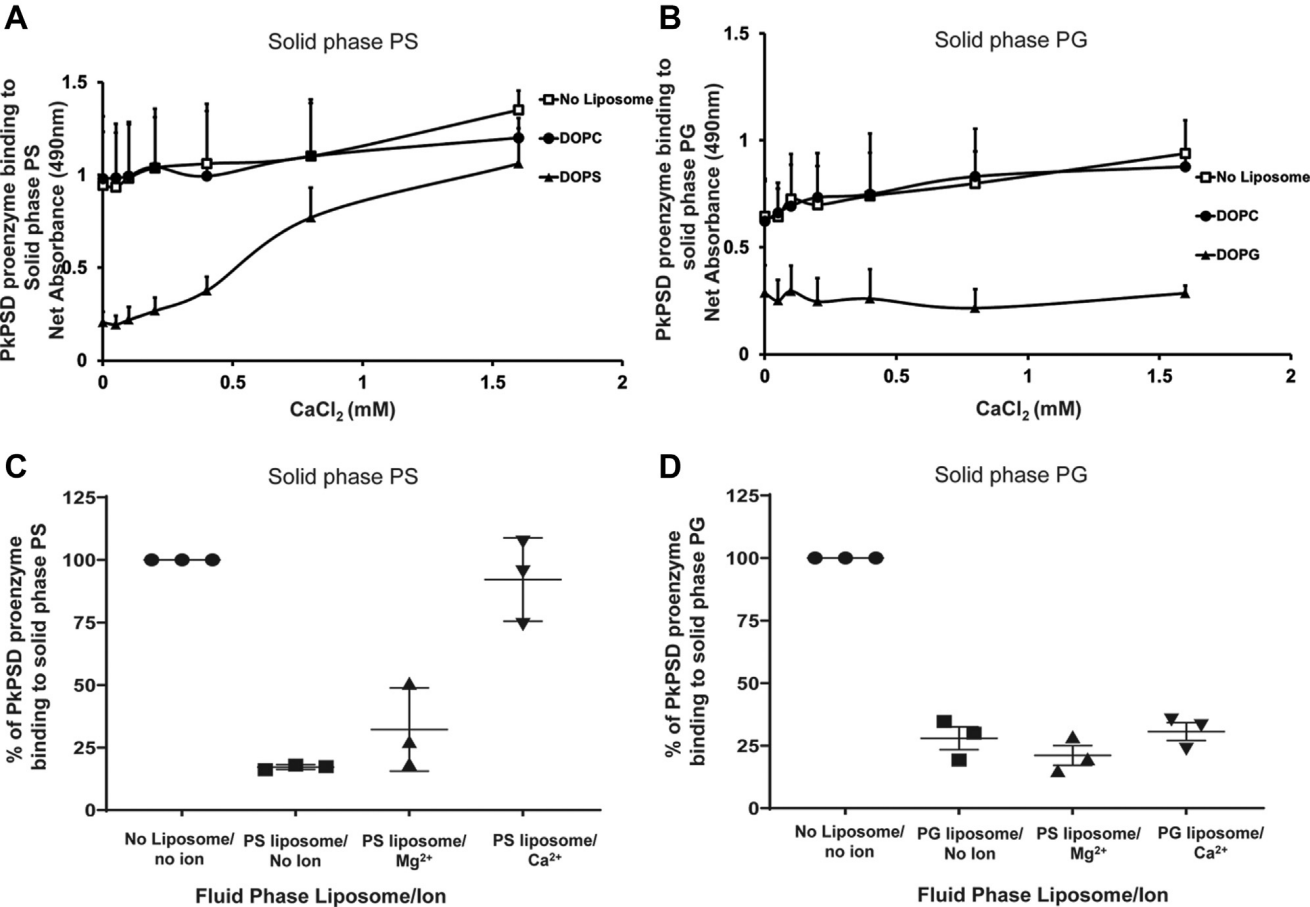
**Calcium ion inhibits ionic interaction of fluid phase PS liposomes, but not fluid phase PG liposomes, with PkPSD.** PkPSD proenzymes (MBPΔ34PkPSD(S308A)) at 3.4 μg/ml were added to the wells precoated with DOPS (*A*) or DOPG (*B*) and incubated at 37 °C, for an hour. As shown in Figure 4, fluid phase liposomes were also added to the wells for their competitive binding to PkPSD proenzymes, over the solid phase lipids. To test if calcium can nullify the competitive fluid phase liposomes binding to PSD proenzyme, various concentrations of calcium ions were also added to the well. The panel shows the strength of the proenzyme binding to the coated DOPS (absorbance values at 490 nm) on the y-axis over the increasing calcium concentrations on the x-axis. Panels *C* and *D* show the effects of magnesium and calcium ion at a fixed concentration (1.6 mM) on the fluid phase competition by liposomes (DOPS or DOPG, for panel *C* or *D*, respectively) and concomitant inhibition of proenzyme binding to the solid phase lipid. The data are from three independent experiments, and values are Means + SD (*A* and *B*) and Means ± SD (*C* and *D*). MBP, maltose binding protein; PG, phosphatidylglycerol; Pk, *Plasmodium knowlesi*; PS, phosphatidylserine; PSD, PS decarboxylase.

Previously, it has been shown that the specific interactions between anionic lipids and proteins can be affected by a local spike in calcium concentrations (32). The data in Figure 6*A* show that increasing concentrations of calcium inhibited the fluid phase PS liposome interaction with the PSD proenzyme. Under these same conditions, fluid phase PS liposomes were no longer able to compete with the solid phase PS for PSD binding. The inhibition of lipid binding by divalent calcium ions appears specific, since divalent magnesium ions failed to show any inhibition (Fig. 6*C*). Interestingly, calcium ions failed to block the fluid phase PG liposome binding to the PSD proenzyme (Fig. 6*D*). The inhibition of PSD proenzyme binding to the solid phase PG was maintained even in the presence of a high concentration of calcium ions as demonstrated in Figure 6, *B* and *D*. The data indicate that the calcium ions regulate PSD proenzyme binding to PS, the activator, but not to PG, an inhibitor of the PSD maturation.

**Figure 7:**
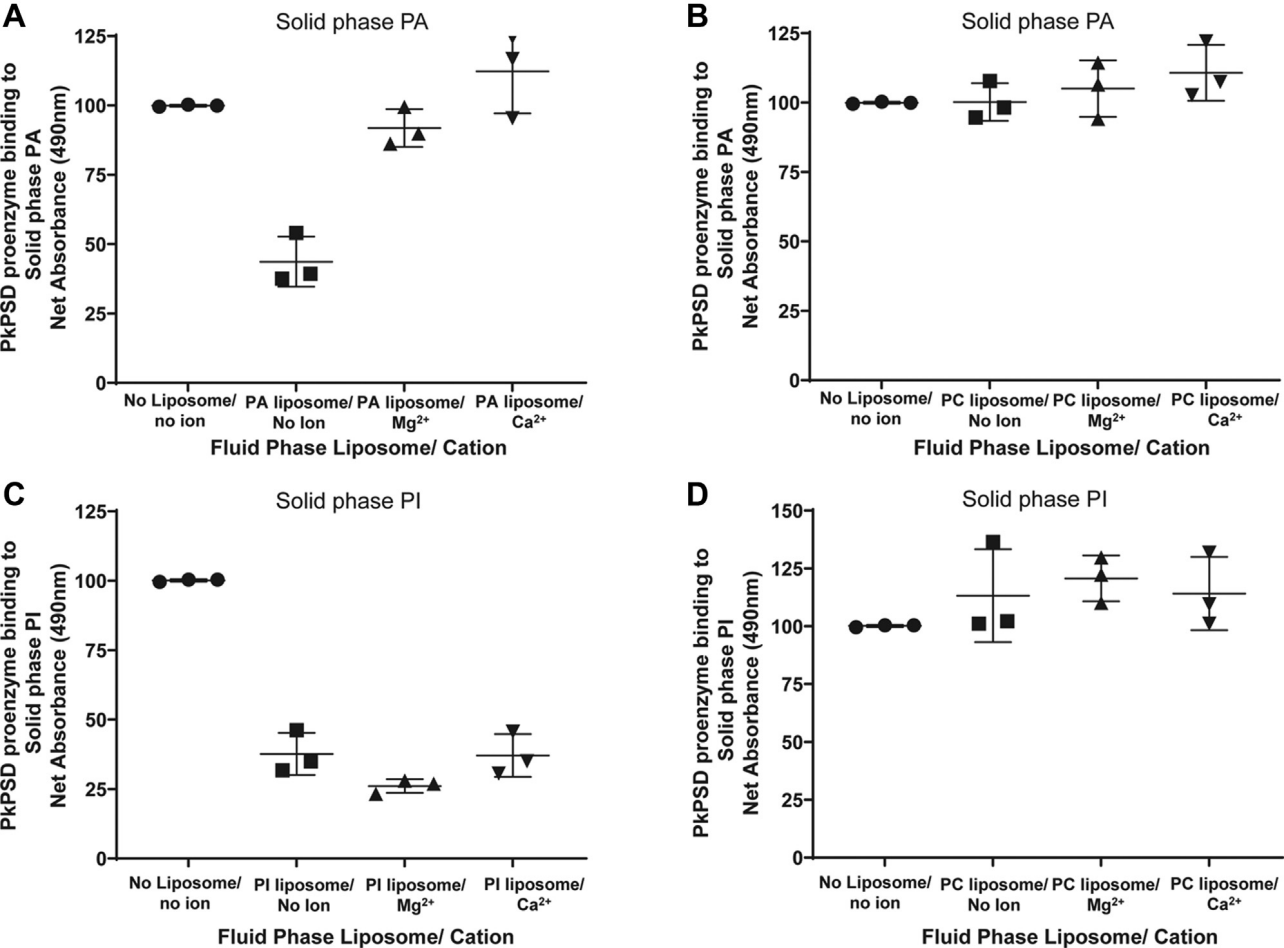
**Other anionic lipids show a different calcium response.** Solid phase binding assays in the presence of fluid phase liposomes and calcium ion were performed as described in Fig, 6. PkPSD proenzymes and fluid phase liposomes consisting of PA (*panel A*) or PC (*panel B*) were added to the solid phase PA. PkPSD proenzymes and fluid phase liposomes, consisting of PI (*panel C*) and PC (*panel D*), were added to the solid phase PI. The data are from three independent experiments, and values are Means ± SD. PA, phosphatidic acid; PC, phosphatidylcholine; PSD, PS decarboxylase; PI, phosphatidylinositol; Pk, *Plasmodium knowlesi*.

In Figure 7, the interactions of calcium ions with the other inhibitory anionic lipids, PA and PI, were also assessed. The fluid phase PA liposome binding to the proenzyme was regulated by calcium ions (Fig. 7*A*), but the fluid phase PI liposome binding was not regulated by calcium ions (Fig. 7*C*). The data presented in Figure 7, *B* and *D* represents the control data of the fluid phase PC liposome that cannot compete with the solid phase PA or PI liposomes for the proenzyme binding, regardless of metal ions. Taken together, the regulation of the anionic lipid binding to proenzyme by calcium ions are not dependent on the mode of activation/inhibition on the PSD processing, but rather on the structures of the anionic lipids. Both PG and PI contain multiple hydroxyl moieties in the polar head group. Next, we asked if calcium inhibition of binding of the PS to the PSD proenzyme could inhibit processing of the proenzyme into mature PSD. To test this, we generated crude *E. coli* cell extracts highly enriched with proenzyme forms of WT PkPSD, after a short IPTG induction, and conducted time course studies to follow the *in vitro* PkPSD maturation reaction.

**Figure 8:**
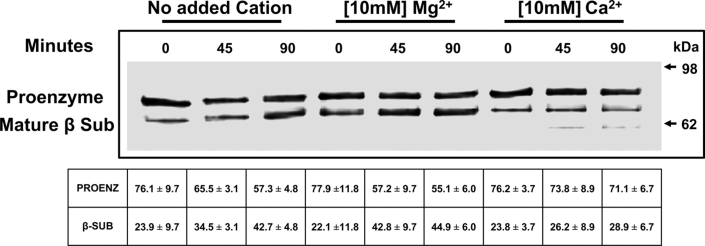
**Calcium ions inhibit *in vitro* maturation of PkPSD proenzyme.***Rosetta DE3* strains harboring pMAL-c2x-MBP-His_6_-*Δ*34PkPSD were induced with 0.3 mM IPTG for a short time (20 min at 37 °C) to ensure the enrichment of the proenzyme forms of MBP-His_6_-*Δ*34PkPSD. Cell-free extracts containing proenzyme forms were diluted to 0.03 μg-protein/μl in the buffer (20 mM Tris–HCl, pH 7.4, 200 mM NaCl, 10 mM β mercaptoethanol, 1 mM EDTA, and 0.1 mg/ml DOPS liposomes) and incubated *in vitro* to allow their maturation (*via* autoendoproteolysis) into active enzymes for 0, 45, or 90 min. Formation of β-subunits is a measure of proteolysis/maturation. Ten millimolar divalent cations (magnesium or calcium ions) were added to the *in vitro* reaction, to assess their roles in the processing. PSD proteins were analyzed by Western blot analysis using primary mouse anti-MBP antibody and secondary goat HRP-conjugated anti mouse antibody. The data are from four independent experiments, and values are Means ± SD. MBP, maltose binding protein; Pk, *Plasmodium knowles*i; PSD, PS decarboxylase.

Figure 8 shows the processing of the WT proenzyme into the mature form after incubation of the cell extracts up to 90 min. During the period, mature β subunit was increased from 23.9% to 42.7% in the control reaction. Mature β subunit was only increased from 23.8 % to 28.9 % in the presence of calcium ions, whereas it was increased from 22.1 % to 44.9 % with magnesium ions, indicating that *in vitro* maturation reaction of the PkPSD proenzyme was inhibited by calcium ions but not by magnesium ions.

### Mapping of the PS-binding domain

**Figure 9:**
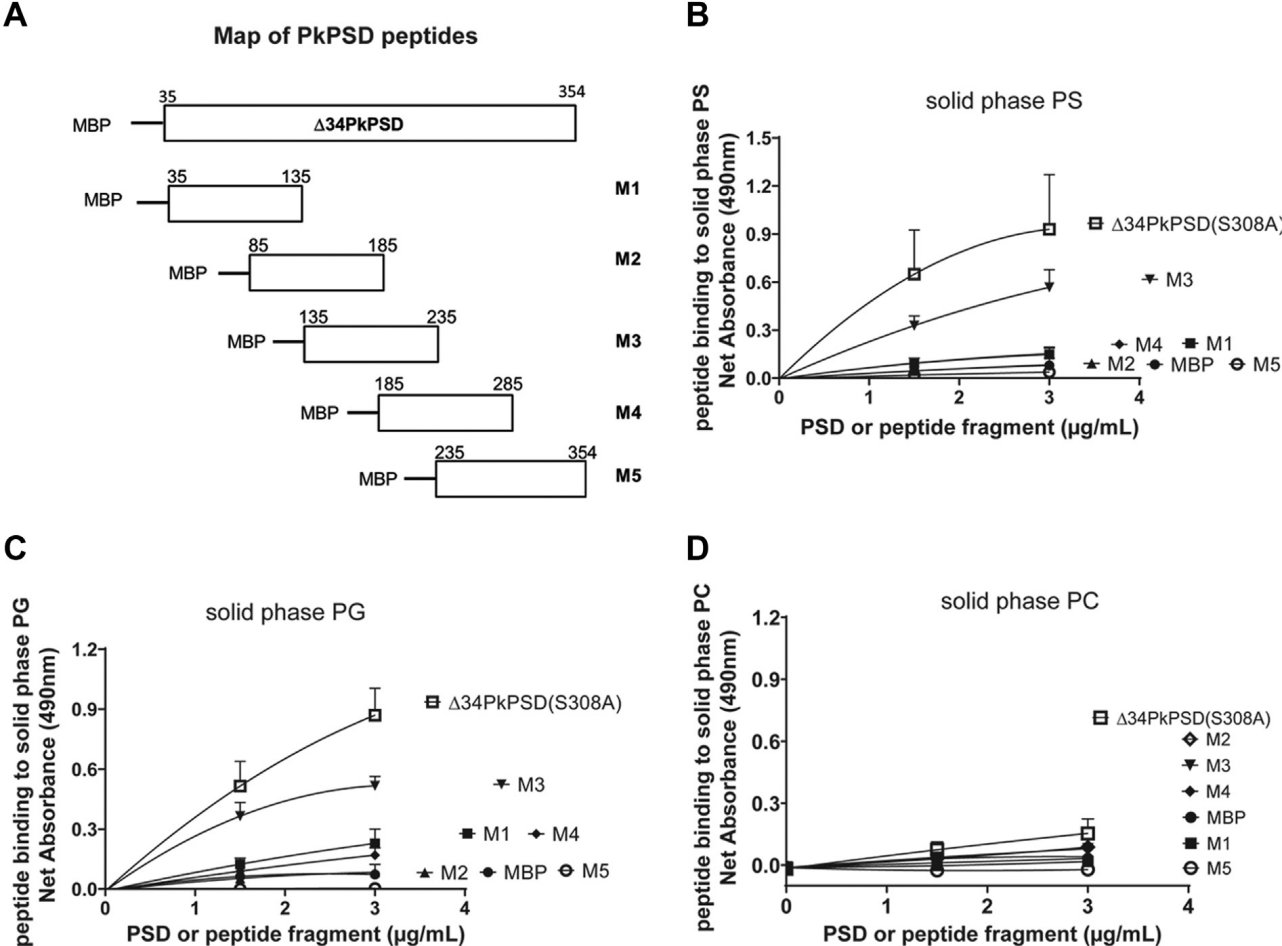
**Identification of a peptide fragment critical for the anionic phospholipid binding.***A*, schematic diagram of the MBP-fused PkPSD peptides. The PkPSD peptides containing 101 amino acids (the most C-terminal fusion peptide contains 119 amino acids) were created by fusing the MBP to the N-terminus of the partial PSD sequences as depicted in (*A*). *PanelsB*, *C*, *and D*, solid phase binding assays were performed with the PSD peptides and PS (*panel B*), PG (*panel C*), or PC (*panel D*) coated onto the wells of the 96-well plates. The PSD peptides at a concentration of 0, 1.7, or 3.4 μg/ml were incubated with lipids precoated the 96-well plate at 37 °C for an hour. PSD peptides bound to the coated lipids on the wells of the plate were detected by ELISA assay using anti-MBP antibody and quantified by measuring absorbance at 490 nm. The data are from three independent experiments, and values are Means + SD. MBP, maltose binding protein; PC, phosphatidylcholine; PG, phosphatidylglycerol; Pk, *Plasmodium knowlesi*; PS, phosphatidylserine; PSD, PS decarboxylase.

**Figure 10:**
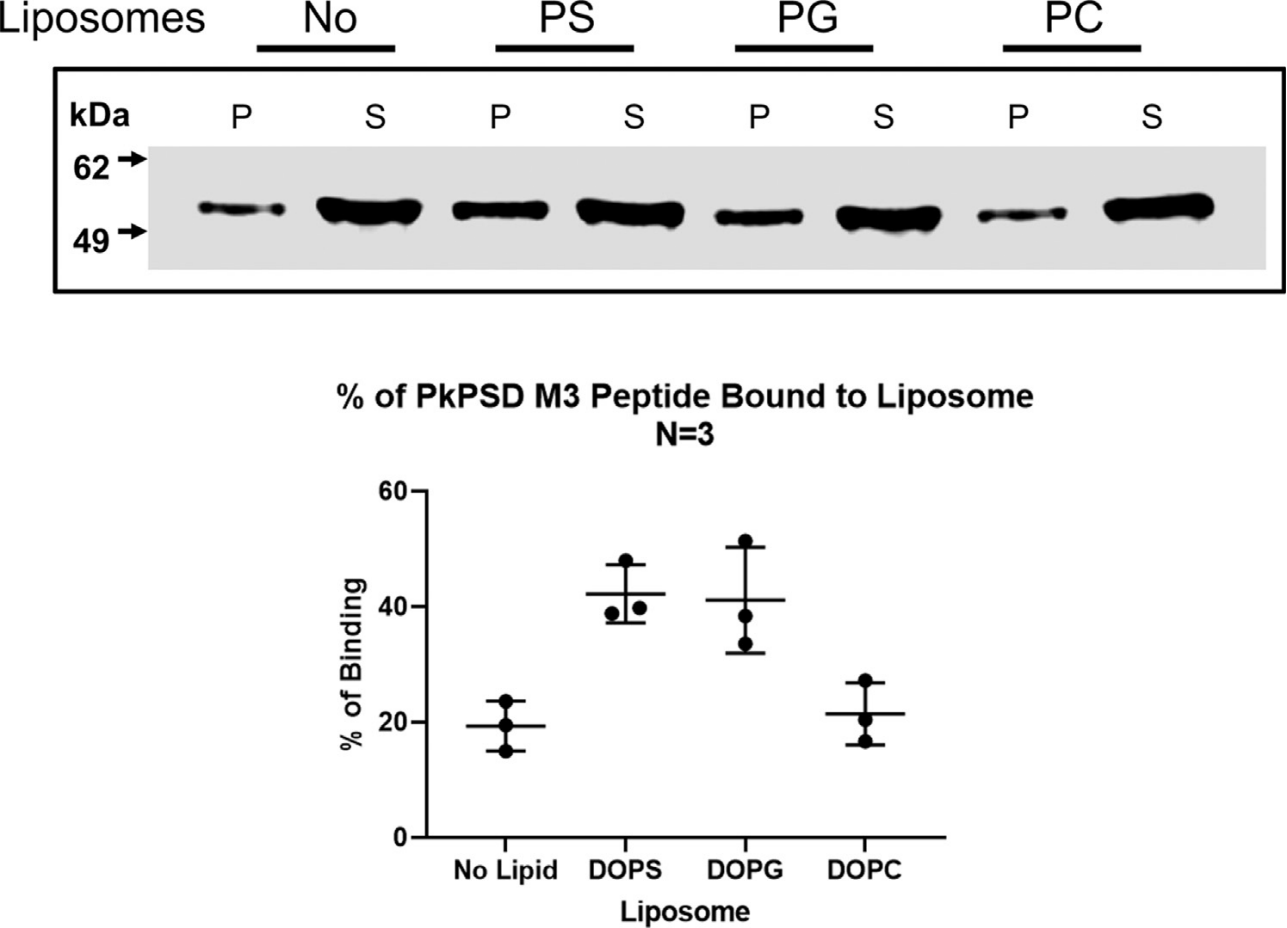
**M3 peptide binds to multilamellar liposomes of PS and PG.** Peptide-lipid binding was performed by incubating affinity-purified MBP-M3 peptide (5 μg/ml) and freshly prepared multilamellar liposomes (0.2 mg/ml) at 37 °C for 45 min with shaking at 150 rpm. The mixtures were centrifuged at 10,000 g for 5 min at 4 °C. The supernatants were carefully transferred to a fresh tube, and the liposome pellets were resuspended in 0.25 ml of buffer. Proteins present in both fractions were analyzed by SDS-PAGE, followed by Western blot analysis, using primary mouse anti-MBP antibody and secondary goat HRP-conjugated, anti-mouse antibody. The data are from three independent experiments and values are Means ± SD. MBP, maltose binding protein; PS, phosphatidylserine; PG, phosphatidylglycerol.

To identify the anionic lipid-binding domains within the PSD proenzyme, partial peptide sequences of the PkPSD proenzyme were created by fusing MBP to the N-terminus of the partial PSD sequences as depicted schematically in Figure 9*A*. Solid phase binding assays were conducted to evaluate the binding activity of the PSD peptides with solid phase PS, PG, or PC, respectively, coated on the wells of a 96-well plate (Fig. 9, *B*, *C*, and *D*, respectively). The data demonstrate that the PSD peptide, designated as M3, could bind to solid phase PS and PG, but only very weakly to the zwitterionic control lipid, PC. Strong binding of the M3 peptide to the anionic lipids was also confirmed by multilamellar liposome sedimentation assays followed by Western blot analysis as shown in Figure 10. More than 40% of the M3 peptides were associated with PS or PG liposomes, following preincubation of the liposomes with the peptides, but less than 20% of the M3 was pelleted after preincubation with either mock or PC multilamellar liposomes.

## Discussion

### Anionic phospholipid binding to the proenzyme for regulation of maturation

Many proteins undergo posttranslational modification to achieve final structure and function. The modifications include proteolytic cleavage and attachment of modifying groups to amino acids through phosphorylation, acetylation, glycosylation, and sulfation, among others (33). PkPSD undergoes specific autoendoproteolytic cleavage between glycine 307 and serine 308 residues (1). Similar cleavages have been reported in other proteins, such as ephitin (34), distroglycan (35), and the G-protein coupled receptor Ig-Hepta (36). In the highly investigated mucin protein (37), a structural constraint has been proposed to trigger proteolytic cleavage. It is currently unknown if PSD cleavage requires a similar structural triggering mechanism. The cleavage within the PSD proenzyme also involves a further modification, the conversion of serine into a pyruvoyl moiety in a concerted reaction (1). In malarial PSD, negatively charged phospholipids have been shown to regulate the processing when the proenzyme was synthesized in an *in vitro* reaction (3). In this report, we investigated how the regulation occurs between the proenzymes and the phospholipid molecules. By using a solid phase binding assay, liposome cosedimentation, and SPR analysis, we demonstrate that the PSD proenzyme and PS, an activator of the maturation, have strong and stable physical interactions. This indicates that binding of the PS to the proenzyme is likely crucial for the processing. Once the proenzyme is converted into the active PSD enzyme, the PSD again binds to PS, as a substrate of the decarboxylase reaction. Recently, two groups independently reported crystal structures of *E. coli* PSD in the absence and presence of PS (38, 39). PS interaction with the mature PSD enzyme was elucidated, wherein the phosphoserine head group interaction and fatty acyl chain interactions occur. The crystal structure of the PSD proenzyme, however, has not been available. The PS-binding mechanism, and PS-binding locus, on the PSD proenzyme might be different from those of the mature PSD.

**Figure 11:**
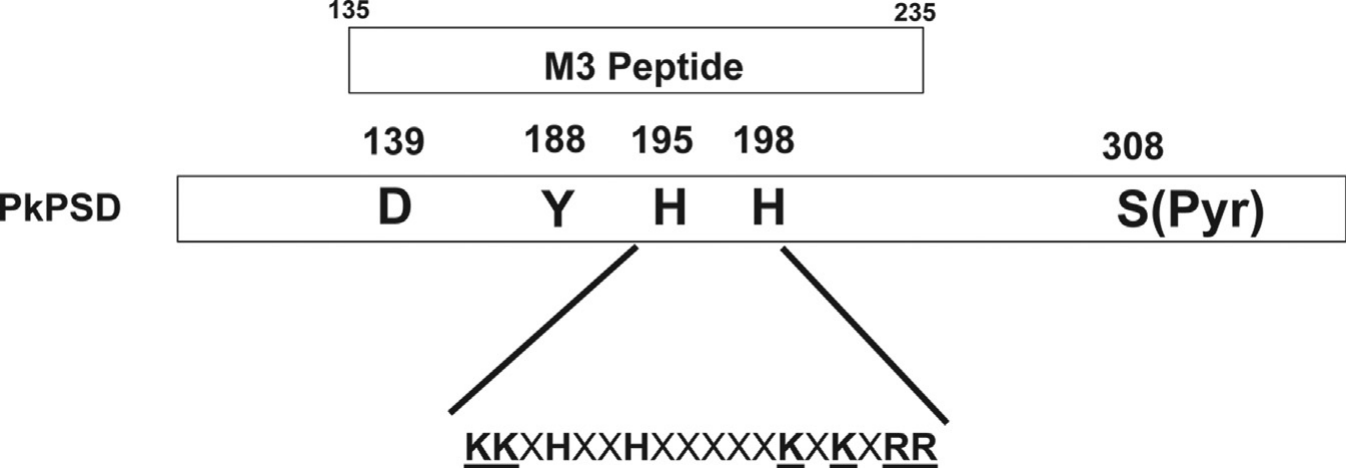
**The PS-binding domain of PkPSD contains a stretch of 8 positively charged amino acids.** M3 peptides contains two of the three amino acids in the active center catalytic triad essential for protease function of the PkPSD proenzyme (139D and 198H) and a highly basic stretch of 18 amino acids encompassing the essential (for decarboxylation) amino acids, 195H and 198H (26). Pk, *Plasmodium knowlesi*; PS, phosphatidylserine; PSD, PS decarboxylase.

**Figure 12:**
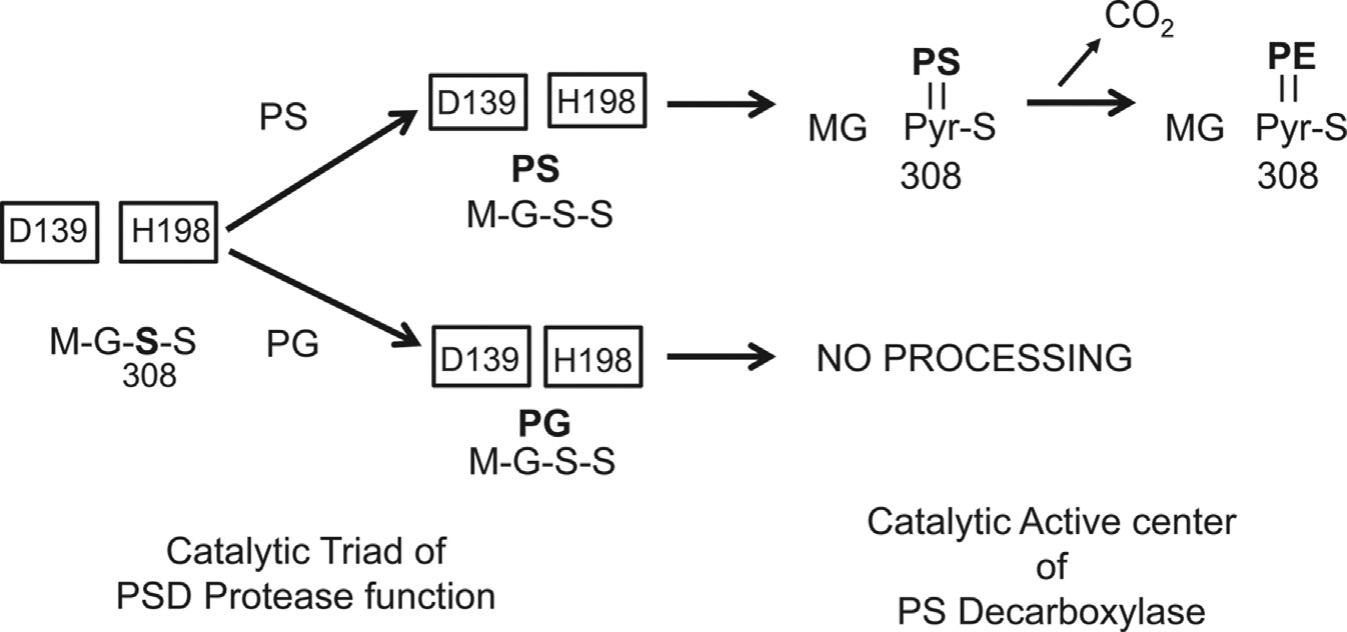
**Model of structural changes at the active center of the PkPSD proenzyme upon PS binding.** Upon PS binding to the basic amino acid sequences on the M3 peptide, the proenzyme undergoes a structural change in the active center resulting in maturation of the PSD proenzyme into an active enzyme consisting of *α* and *β* subunits. PG binding to the proenzyme prevents conversion of the proenzyme to mature PSD enzyme. PG, phosphatidylglycerol; Pk, *Plasmodium knowlesi*; PS, phosphatidylserine; PSD, PS decarboxylase.

The lipid-binding locus within the PkPSD proenzyme was investigated by peptide mapping analysis. As shown in Figures 9 and 10, the M3 PkPSD peptide contains the anionic lipid-binding sites. The M3 PSD peptide contains 101 amino acids which span from amino acids 135 to 235 of the PkPSD proenzyme. Interestingly, the M3 peptide contains 139D and 198H, which were previously identified as essential for the PSD maturation as constituents of the catalytic triad required for the serine protease activity (26). Another highly conserved amino acid 195H which is located near 198H in the M3 peptide was previously found to be essential for decarboxylase activity, although it was not required for serine protease function of the PSD proenzyme (26). The presence of amino acids forming the catalytic triad and high local content of positively charged amino acids, within the M3 peptide, indicate that a subfragment of the M3 peptide constitutes a crucial component in the lipid binding. The 18 amino acid peptide sequence flanking the essential residues, 195H and 198H, contains six positively charged amino acids, which can be involved in the ionic interactions with the negatively charged anionic phospholipids (Fig. 11). Figure 12 is the proposed model suggesting the structural changes occurring at the catalytic active center of the PkPSD proenzyme during the maturation process. The maturation process is inhibited by PG binding to the proenzyme. Upon PS binding to the basic amino acid residues present within this locus in the M3 peptide, the proenzyme undergoes structural change at the active center resulting in conversion to an active enzyme, with a pyruvoyl prosthetic group on the α subunit. With this latter structural change, the active site of the PkPSD active enzyme binds to PS *via* a Schiff base formation between the primary amine of the PS head group and the α-carbonyl carbon of the pyruvoyl residue (1). Subsequent decarboxylation generates the product, PE, in Schiff base linkage to the PkPSD enzyme. A hydrolysis reaction across the Schiff base regenerates the pyruvoyl prosthetic group and releases PE from the active site.

### Mechanism of proenzyme interaction with anionic lipids depends on ionic strength of medium

We investigated the interaction of the proenzyme with the lipid molecules. Since negatively charged lipids can participate in ionic interactions, increasing concentrations of NaCl were added to the binding reactions to assess the likelihood of ionic interactions as a mechanism of association. Notably, peptide binding was weakened under high salt conditions consistent with a binding mechanism requiring ionic interactions. Lipid and protein interactions are crucial for many cellular activities. Two types of lipid and protein interactions have been very well studied. One is comprised of many types of lipid-binding domains such as C2, PH, FYVE, and ENTH (40). The other has been proposed for the proteins with polybasic amino acid stretches interacting with the anionic lipids (41, 42). The T cell receptor–CD3 complex is a membrane receptor crucial for adaptive immunity and its activation is controlled by ionic interactions between the negatively charged lipids in the inner leaflet of the plasma membrane and a positively charged cytoplasmic domain of the protein. Calcium influx was shown to disrupt the binding and to lead to the activation of the T cell receptor (32). Here, we show that calcium ions play a role in the ionic interaction of PkPSD and the anionic lipid, PS. Interestingly, the binding of proenzyme to PS liposomes, but not to PG liposomes, was specifically blocked by calcium ion. The blocking of the binding of PS to proenzyme by calcium also affected the *in vitro* processing of the WT PkPSD proenzymes into the mature forms. The differential mode of the calcium ion effect on PS, an activator of the proenzyme processing, but not on PG, as an inhibitor of processing, implies that activation of PkPSD processing can be regulated by changes in local calcium concentrations.

### Implications for the inhibition of PSD activity by targeting PSD maturation

Previously, we reported two highly sensitive systems for measuring PSD activity (43, 44). The assays are based on the fluorescent compounds, DSB-3, or DAB/BME, forming a fluorescent adduct with the enzyme reaction product, PE, at an elevated pH. These assays are amenable to high-throughput screening for PSD inhibitors (45). The inhibition of the PSD activities can occur either by disrupting the maturation of the proenzyme into the active enzyme or disruption of the PSD enzyme reaction. Hence, blocking or enhancing the steps of the PS lipid interaction with the proenzyme can be a major target to regulate the PSD processing and ultimately its catalytic activity, which has been suggested to be a target for animicrobials and anticancer therapies (8, 20, 21, 22, 45).

## Experimental procedures

### Materials

All chemicals for bacterial growth media were purchased from Sigma and Thermo Fisher Scientific. All phospholipids used in this study were purchased from Avanti Polar Lipids. Reagents for quantifying protein were from Bio-Rad. Corning 96-well clear polystyrene microplates (CLS3695) were from Sigma-Aldrich. Materials and equipment for SPR analysis was purchased from Nicoya.

### Construction of pMAL vectors harboring sequences encoding for PkPSD peptides

303 bp of DNA sequences encoding partial PkPSD peptides (M1:1–300, M2:150–450, M3:300–600, M4:450–750, and M5:600–960) of *Δ34PkPSD* were commercially synthesized and inserted into pMAL-c4x (Genscript). The resulting constructs were transformed into *Rosetta DE3* strains by selective growth in LB plates containing ampicillin and chloramphenicol.

### Expression and purification of MBP -Δ34PkPSD(S308A)

Expression of MBP-Δ34PkPSD(S308A) in *E*. *coli* was performed as described (26). Briefly, a *Rosetta DE3* strain harboring a pMAL-c2x-Δ34PkPSD(S308A) plasmid vector was grown to saturation overnight in 1 L of LB medium with 0.2% glucose, ampicillin (100 μg/ml), and chloramphenicol (34 μg/ml), then diluted 100-fold, and grown to A600 ∼0.5 at 37 °C. Expression of MBP-His_6_-Δ34PkPSD was induced by the addition of 0.3 mM IPTG for 2 h at 37 °C. The cells were harvested by centrifugation (4000*g*, 20 min, 4 °C) and washed by resuspension in water and recentrifugation. The cells were resuspended in 25 ml of disruption buffer (20 mM potassium phosphate, pH 7.4, 200 mM NaCl, 1 mM EDTA, and 10 mM β-ME), flash frozen in a dry ice-ethanol bath, stored overnight at −20 °C, and subsequently thawed on ice water. Cell extracts were obtained by sonication (15 s burst at 30 % amplitude using a Fisher Sonic Dismembrator 500, performed 8 times, interrupted by 30 s cooling intervals, using an ice water bath), followed by centrifugation at 20,000*g* for 20 min, at 4 °C. MBP-Δ34PkPSD(S308A) was purified from the resultant supernatants by amylose column affinity chromatography using methods described in the instruction manual from New England Biolabs (#E8200S). Briefly, the cell extracts were further diluted 5-fold in disruption buffer and applied to an amylose affinity column (∼10 ml). The column was washed with 6 ml aliquots of the disruption buffer, 11 times. MBP-Δ34PkPSD(S308A) proteins were eluted with the disruption buffer containing 10 mM maltose and 20 fractions of 1.2 ml each were collected. The fractions containing the MBP-Δ34PkPSD(S308A) proteins were identified by SDS-PAGE, followed by Coomassie staining of the gel and Western blot analysis using anti-His_6_ antibody.

### Solid phase binding assay

PkPSD binding to phospholipids was conducted as described with some modifications (46). Phospholipids at 50 μM in ethanol were prepared from phospholipid solutions in chloroform (10 mg/ml). The lipids in chloroform were dried under a stream of nitrogen gas using an Organomation nitrogen evaporator. Once dried, the resultant lipid film was resuspended in ∼50 μl of ethanol. 1.25 nmol (25 μl of 50 μM solution) of the phospholipids were plated into the well of the 96-well plate and dried under a stream of room air for 90 min. A blocking solution (3% bovine serum albumin, 100 mM NaCl, and 10 mM potassium phosphate, pH 7.4) was added to each well coated with the phospholipid, and the plate was incubated at 37 °C for 1 h. The blocking solution was removed, and each well was washed 3 times with 150 μl of the washing buffer (100 mM NaCl and 10 mM potassium phosphate, pH 7.4). Binding reactions were performed by the addition of 50 μl of MBP-Δ34PkPSD(S308A) in the blocking solution at various concentrations (0 ∼ 3.2 μg/ml) and incubation at 37 °C for 1 h. When fluid phase lipid competition analysis for PSD binding to solid phase lipids was conducted, various concentrations of fluid phase unilamellar liposomes (0–1.25 nmol) were also added to the well. The protein solutions (and the optional fluid phase liposomes when added) were discarded, and each well was washed at least 3 times with 150 μl of the washing buffer. The proteins bound to the coated lipids were detected with anti-MBP monoclonal antibody (NEB, E8032S, diluted by 30,000-fold in the blocking buffer) and then HRP-IgG–conjugated secondary antibody (Sigma Aldrich, cat#12-349, diluted by 15,000-fold in the blocking buffer). The binding capacity was detected by peroxidase assay using an orthophenylenediamine substrate tablet and H_2_O_2_. The color reaction was measured by reading that absorbance at 490 nm using a 96-well plate reader.

### Liposome cosedimentation assay

Liposome cosedimentation assays were conducted as described (8, 47). In short, 5X stock solutions of multilamellar liposomes (1 mg/ml) were prepared from lipid solutions in chloroform (10 mg/ml), purchased from Avanti. Lipids in chloroform (40 μl) were transferred into a glass tube and dried under a stream of nitrogen gas. Once dried, the lipid film was resuspended in 100 μl of methanol and dried again using a stream of nitrogen gas, to remove any residual chloroform. The dried lipids were resuspended in 400 μl of 0.1 M NaCl and 10 mM potassium phosphate buffer, pH 7.4, mixed with a vortex mixer, and hydrated at 37 °C, for 30 min to create multilamellar liposomes. A 50 μl aliquot of multilamellar liposomes was mixed with 150 μl of the bovine serum albumin blocking solution (0.83 mg/ml) in 0.1 M NaCl and 10 mM potassium phosphate buffer, pH 7.4. The binding reaction was initiated by the addition of 40 μl of PSD protein solution into the tube. The reaction was incubated at 37 °C for 40 min, with shaking at 150 rpm. Following the incubation, the tubes were centrifuged at 10,000*g*, for 5 min at 4 °C, to recover sedimentable multilamellar liposomes (P). The supernatant was transferred to a new tube. The pellet was resuspended in equal volume of the buffer solution. The liposome-bound PSD (the pellet) and the unbound PSD (the supernatant) were detected by SDS-protein gel analysis, combined with Western blot analysis using a mouse anti-MBP antibody and a goat HRP-conjugated anti mouse antibody. The bound PSD was quantified by comparing the protein band intensities from the pellet and the supernatant fractions using ImageJ software (NIH).

### SPR analysis

The SPR experiments were performed using an OpenSPR (Nicoya) equipped with a research-grade LIP-1 sensor chip. Unilamellar liposomes (DOPS, DOPG, or DOPC at 0.5 mg/ml in in 10 mM potassium phosphate buffer, pH 7.4 and 100 mM NaCl) were prepared by sonication and were immobilized through the interaction between acyl chain of the lipid and the hydrophobic chains on the sensor chip at a density of 2000 ∼ 5000 RU on channel 2. Channel 1 was left blank to serve as a reference surface. Both channels were blocked with an injection of 25 mg/ml glucose. To collect kinetic binding data, MBP-Δ34PkPSD(S308A) in 10 mM Hepes, 100 mM NaCl, pH 7.4, was injected over the two flow cells at concentrations of 140 nM, 420 nM, 1.3 μM, 3.9 μM, and 7.8 μM at a flow rate of 20 μl/min and at a temperature of 20 °C. The complex was allowed to associate and dissociate for 240 and 360 s, respectively. The surfaces were regenerated with an injection of 5 mM NaOH at a flow rate of 150 μl/min. Binding kinetics data were collected on a simple 1:1 interaction model using the TraceDrawer software (Nicoya).

## Data availability

All data described are contained within the article.

## Conflict of interest

The authors declare that they have no conflicts of interest with the contents of this article.
